# Gender differences in the dissection properties of ascending thoracic aortic aneurysms

**DOI:** 10.1093/icvts/ivac068

**Published:** 2022-03-14

**Authors:** Jianhua Tong, Mieradilijiang Abudupataer, Xiaojuan Xu, Zhi Zhang, Jun Li, Hao Lai, Chunsheng Wang, Kai Zhu

**Affiliations:** 1 Institute for Biomedical Engineering and Nano Science, Shanghai East Hospital, Tongji University School of Medicine, Shanghai, China; 2 Department of Cardiac Surgery, Zhongshan Hospital, Fudan University, and Shanghai Institute of Cardiovascular Diseases, Shanghai, China; 3 Department of Pathology and Pathophysiology, Tongji University School of Medicine, Shanghai, China

**Keywords:** Ascending thoracic aortic aneurysm, Aortic dissection, Gender differences, Delamination strength, Age

## Abstract

**OBJECTIVES:**

Presentation, management and outcomes in the aortic dissection (AD) of ascending thoracic aortic aneurysm (ATAA) differ in gender and age. The purpose of this study is to investigate the dissection properties of male and female ATAAs.

**METHODS:**

Peeling tests were performed to quantitatively determine the delamination strength and dissection energy of 41 fresh ATAA samples (22 males and 19 females) in relatively young (≤65 years) and elderly (>65 years) patients. The delamination strength of the ATAAs was further correlated with patient ages for males and females. The histological investigation was employed to characterize the dissected morphology.

**RESULTS:**

For elderly patients, circumferential and longitudinal delamination strengths of the female ATAAs were statistically significantly lower than those of the males (circumferential: 31 ± 6 vs 42 ± 6 mN/mm, *P* < 0.01; longitudinal: 35 ± 7 vs 49 ± 10 mN/mm, *P* = 0.02). No significant differences were found in the delamination strength between males and females for relatively young patients. The circumferential and longitudinal delamination strengths were significantly decreased and strongly correlated with patient ages for females. However, these correlations were not present in males. Dissection routes propagated in the aortic media to create ruptured surfaces for all specimens. Peeling tests of the male ATAAs generate rougher surfaces than females.

**CONCLUSIONS:**

There is a higher propensity of AD occurrence for the elderly females as compared to males with matched ages. Surgeons should be cognizant of the risk of AD onset later in life, especially in females.

**Figure:**
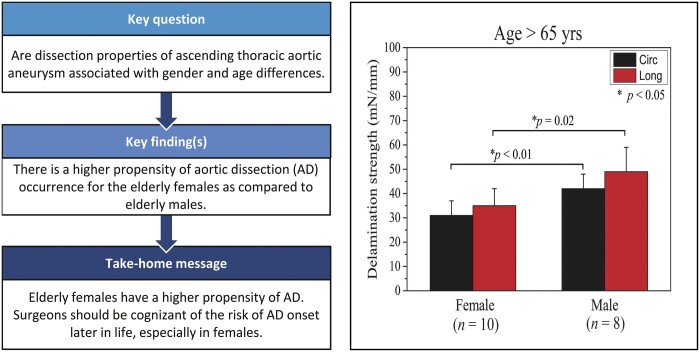

## INTRODUCTION

Ascending thoracic aortic aneurysm (ATAA) is a clinically significant pathology associated with high morbidity and mortality [1, 2]. The most common complication of the ATAA is aortic dissection (AD), leading to high mortality rate if patients are not diagnosed early and surgically treated [3, 4]. AD represents a devastating separation of elastic layers when haemodynamic loads exceed adhesive strength between layers, and therefore, it involves a complex series of biomechanical events [5–7]. Biomechanical analysis is helpful to characterize emerging behaviours of AD and to determine delamination strength as a biomarker for the risk of dissection [6–10].

Previous studies indicated that the risk of dissection for ATAAs was gender related [1, 11–13]. Although the incidence of AD was higher in males, females tended to be older when dissection occurred [14]. This may be related to worse biomechanical properties of elderly females’ aortas. As a biomechanical indicator, greater aortic stiffness was associated with greater aneurysmal growth rate in females and, thus, contributed to enhancing the risk of tissue stratification [13, 15]. However, it remains unknown whether the elderly females have biomechanically worse aortas that require lower forces to dissect. There is a pressing need to thoroughly investigate differences in the delamination strength of ATAA tissue between males and females from a biomechanical point of view.

The present study focuses on 3 aims: (i) we experimentally investigate dissection properties of fresh ATAA samples acquired from patients using peeling test; (ii) we compare dissection properties of males with females in relatively young (≤65 years) and elderly (>65 years) patients and correlate the delamination strength of ATAAs with age for male and female patients, respectively; and (iii) we characterize morphology of the dissected surfaces of ATAAs in different genders.

## MATERIALS AND METHODS

### Ethics statement

Human aortic specimens were utilized under approvals of Zhongshan Hospital, Fudan University Ethics Committee (no. B2020-158) in accordance with the Declaration of Helsinki. Written informed consent was obtained from all patients before participation.

### Tissue acquisition

**Figure 1:**
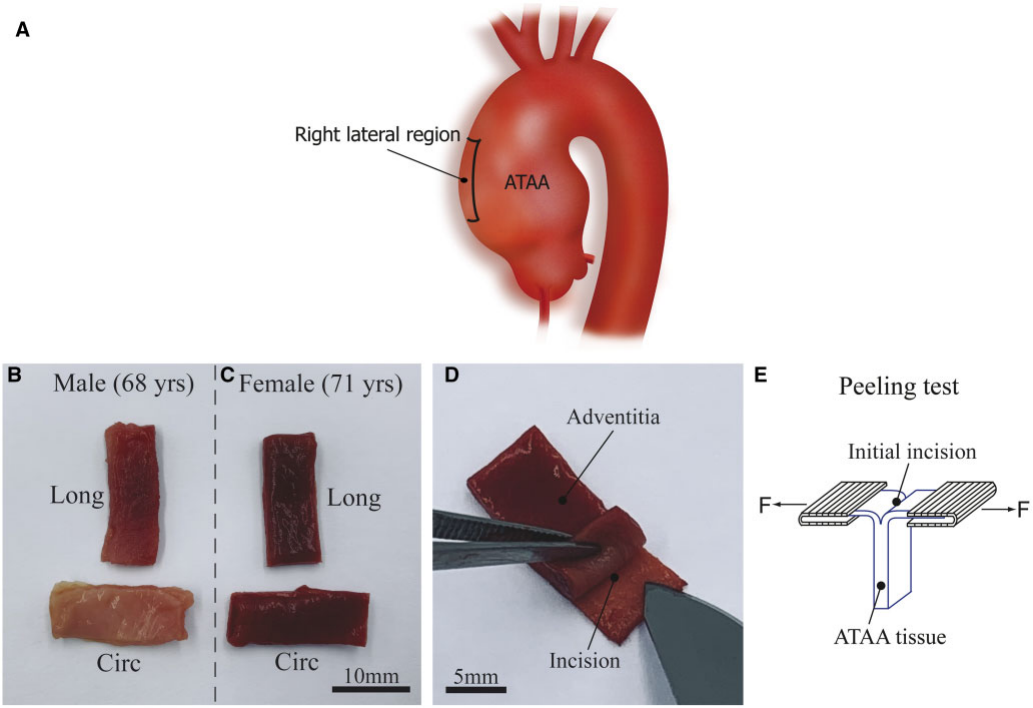
Schematic illustration of specimen cutting and preparation for the peeling test. (**a**) Tissue samples are cut from right lateral region of the ascending thoracic aortic aneurysms. (**b**) Two rectangular strips are extracted from the circumferential and longitudinal directions of the individual fresh ascending thoracic aortic aneurysm samples (donors: male, 68 years and female, 71 years), (**c**) an initial incision is given to the rectangular strip of the ascending thoracic aortic aneurysm tissue and (**d**) 2 free flaps (glued with sandpapers) are further mounted on the testing machine to perform peeling test. Circ and Long denote the circumferential and longitudinal directions, respectively.

This study was approved by Ethics Committee of Zhongshan Hospital, Fudan University, Shanghai (Approval Letter No. B2020-158) in accordance with the Declaration of Helsinki. Human aortic tissues were harvested from 22 male (age, 57 ± 10 years) and 19 female patients (age, 61 ± 8 years) who were diagnosed with the concomitant ATAAs and underwent elective ascending aorta surgery at the Department of Cardiac Surgery of Zhongshan Hospital, Fudan University. Echocardiography was used to characterize the aortic valve morphology, and ascending aortic diameter was measured by computerized tomography prior to surgery. All samples were cut from right lateral region of the ascending aortic aneurysms (Fig. 1a). The acquired specimens were stored in phosphate-buffered saline (PBS) solution at 4°C and sent for mechanical testing within 6 h after retrieval.

### Specimen preparation

The ATAA tissue was cut into rectangular pieces with a dimension of 18.0 mm × 6.0 mm (length × width) along the circumferential and longitudinal directions for both male and female groups (Fig. 1b). Thickness of the specimen was measured optically using a video extensometer [16, 17]. To prepare for the peeling test, each rectangular strip was given an initial cut with an incision of about 3.0 mm in length to better control the initiation of the tissue failure (Fig. 1c). Hence, 2 free flaps were produced for mounting into the testing machine. To avoid the slippage of the specimens during peeling, rectangular pieces of sandpaper were glued with superadhesive gel at both sides of the flaps. The schematic illustration of the peeling test is shown in Fig. 1d. All specimens were moistened with PBS prior to testing.

### Peeling test

Peeling tests were performed on a PC-controlled, screw-driven high-precision tensile testing machine, which is adapted for small biological materials (XJ810-10N, Xiangjie Inc., Shanghai, China). Schematic experimental setup of the peeling test can be referred to in a previous study [18]. Once both free flaps of the prepared specimen were clamped in the testing machine, the individual peeling test was carried out under the prescribed extension rate (i.e. 1 mm/min). The corresponding resisting forces were documented. Note that the peeling tests were conducted in PBS solution at 37.0 ± 1.0°C that was controlled by a heater-circulation unit (Ecoline E 200, Lauda, Lauda-Konigshofen, Germany).

### Data analysis

Force/width value is the ratio between the documented resisting force and the width of the strip specimen that is measured before the peeling test. The mean value of force/width was defined as delamination strength (*S*_d_) of the specimen. To calculate the mean force/width, we fitted the experimental data of the individual tests to horizontal lines in the force/width-dissection path graph (OriginPro 9.0). Subsequently, the force/width values were further averaged across specimens to obtain the arithmetic mean force/width values *F*_pc_ and *F*_pl_ in the circumferential and longitudinal directions, respectively.

In addition, dissection energy per reference area *W*^dissect^ during the (circumferential c and longitudinal l) peeling (p) was calculated by subtracting the elastic energy *W*_elastic_ from the external work *W*_ext_, i.e.

(1) $$W_{\text{pc}}^{\text{dissect}}=\left(W_{c}^{\text{ext}}-W_{pc}^{\mathrm{elastic}}\right)/L_{\text{pc}},W_{\text{pl}}^{\text{dissect}}=\left(W_{l}^{\text{ext}}-W_{\text{pl}}^{\text{elastic}}\right)/L_{\text{pl}},$$

where *L*_pc_ and *L*_pl_ denoted the reference lengths of the strips in the circumferential and longitudinal directions, respectively. Details of calculating the elastic energy and external work can be referred to in previous studies [19, 20].

All quantitative values were reported as mean ± standard deviation. The Shapiro–Wilk and Kolmogorov–Smirnov tests were utilized to determine the data normality. Comparisons of the delamination strength or the dissection energy between male and female groups were performed using one-way analysis of variance followed by the Holm-Sidak test for *posthoc* analysis. A *P*-value of <0.05 determined significance. The statistical analyses were performed using the SPSS statistical package version 21.0 (SPSS Inc./IBM, Chicago, IL).

### Correlating delamination strength with patient age

The quantified delamination strength (i.e. mean force/width) of individual specimen was plotted as a function of patient age for males and females. To fit the scatter data points, a linear regression model in the optimization toolbox of OriginPro 9.0 was utilized. Correlation was evaluated with the Pearson’s rank correlation coefficient *R*. A *P*-value of <0.05 determined statistical significance. A correlation was considered as strong for *R* > 0.60.

### Histology

After mechanical testing, the specimens were fixed in neutral buffered formaldehyde solution (pH 7.4) and then embedded in paraffin. The paraffin blocks were cut at 5-μm section on a Leica RM2126 microtome (Leica, Shanghai, China) and stained with Masson after deparaffinization and hydration. Subsequently, the sections were examined under a Leica DM2500 microscope (Leica Microsystems, Germany) to investigate the morphology of the dissected surfaces.

## RESULTS

### Clinical characteristics

In the present study, 41 samples (22 males and 19 females) were harvested and tested. Clinical characteristics of patients are shown in Table 1. In summary, 24% of patients were elderly females (72 ± 5 years). Among these female patients, the percentage of the bicuspid aortic valve was 20%. There was no statistically significant difference in the mean ascending aortic diameter between the elderly females and the elderly males (52.2 ± 9.0 vs 51.50 ± 4.96 mm, *P* = 0.85). However, the mean sinus diameter of the elderly females was significantly smaller than that of the elderly males (37.70 ± 7.86 vs 45.00 ± 6.16 mm, *P <* 0.05).

**Table 1:** Clinical characteristics of the 41 patients in the present study

Characteristic	Female ≤65 years (*n* = 9)	Female >65 years (*n* = 10)	Male ≤65 years (*n* = 14)	Male >65 years (*n* = 8)
Age, years, mean ± SD	55 ± 6	72 ± 5	52 ± 8	70 ± 3
Tricuspid aortic valve, %	22.2	80	28.6	62.5
Bicuspid aortic valve, %	77.8	20	71.4	37.5
Hypertension, %	44.4	80	35.7	37.5
Diabetes, %	0	10	7.1	0
Ascending aortic diameter, mm, mean ± SD	51.22 ± 6.52	52.20 ± 8.99	49.64 ± 7.11	51.50 ± 4.96
Sinus dilation diameter, mm, mean ± SD	39.44 ± 11.96	37.7 ± 7.86	45.93 ± 7.48	45.00 ± 6.16
Aortic regurgitation (≥moderate), %	22.2	10	14.3	12.5
Aortic stenosis (≥moderate), %	33.3	70	50	50

SD: standard deviation.

### Peeling test

**Figure 2:**
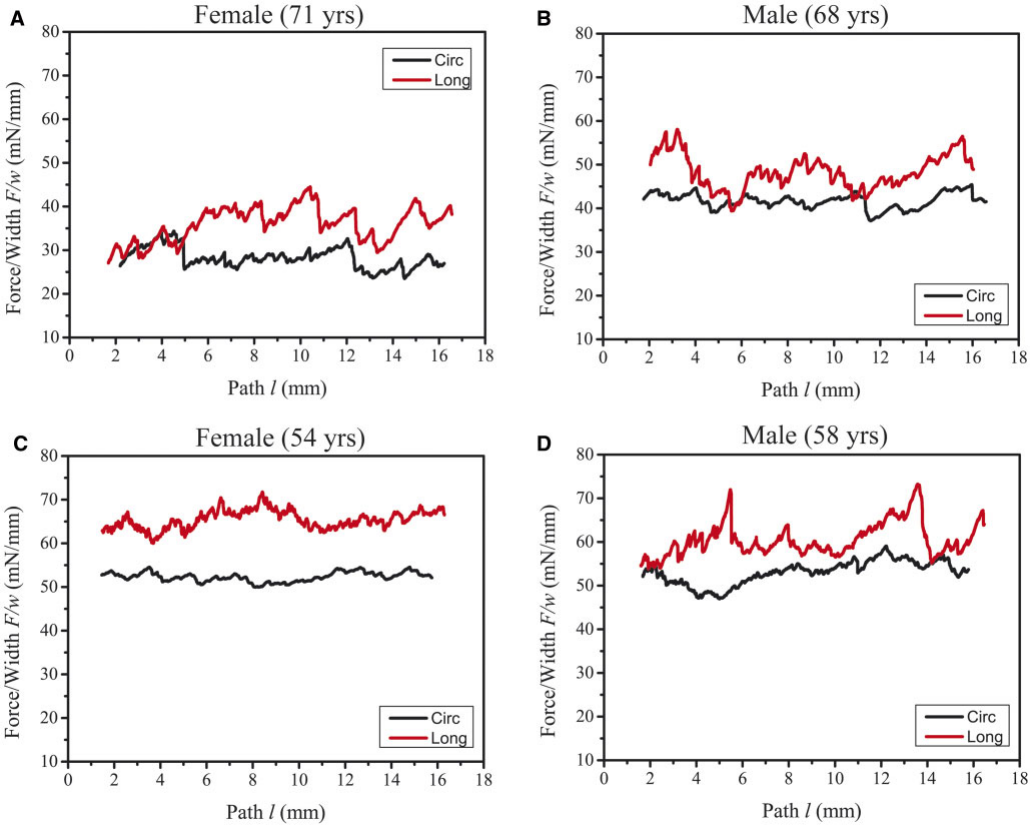
Representative plots of force/width versus the dissection path in the circumferential and longitudinal peeling tests of (**a** and **b**) elderly female and male patients and (**c** and **d**) relatively young female and male patients.

**Figure 3:**
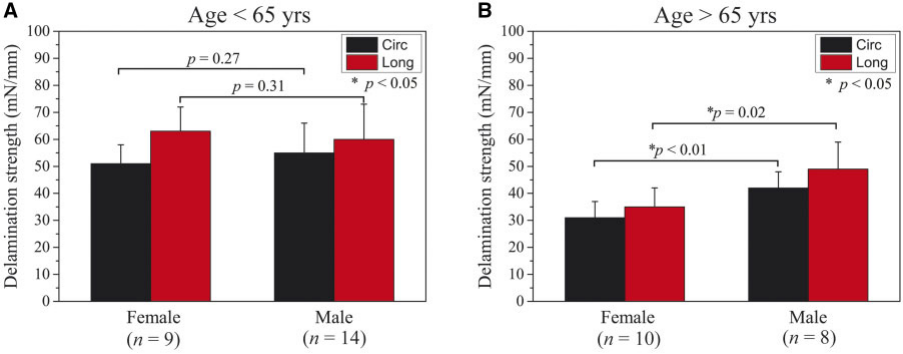
Column plots (mean values and standard deviations) of the measured delamination strength for males and females in the relatively young (≤65 years) and elderly (>65 years) patient groups. The delamination strength values quantified in the circumferential and longitudinal peeling of the elderly female ascending thoracic aortic aneurysms are statistically significantly higher than those of the elderly males. Circ and Long denote the circumferential and longitudinal directions, respectively.

Representative plots of force/width versus dissection path of males and females with different ages are shown in Fig. 2a–d. The black and red jagged curves characterized the resisting performance of the fresh ATAA tissue during the peeling tests in the circumferential and longitudinal directions, respectively. A higher standard deviation was observed in the longitudinal peeling than in the circumferential peeling for both males and females. Column plots of the measured delamination strength for males and females in relatively young (≤65 years) and elderly (>65 years) patient groups are shown in Fig. 3. The Shapiro–Wilk and Kolmogorov–Smirnov tests showed that the quantified delamination strength values were normally distributed. The quantitative results for the different sample groups are summarized in Table 2. For patients <65 years, there were no statistically significant differences in the delamination strength between males and females in the circumferential (*P* = 0.27) and longitudinal (*P* = 0.31) directions (Fig. 3a). For the elderly patients, however, delamination strength to peel apart the female ATAAs was statistically significantly lower than those of the males (circumferential: 31 ± 6 vs 42 ± 6 mN/mm, *P* < 0.01; longitudinal: 35 ± 7 vs 49 ± 10 mN/mm, *P* = 0.02; see Fig. 3b).

**Table 2:** The measured delamination strength values (mN/mm) for the male and female ascending thoracic aortic aneurysm tissues in the circumferential and longitudinal peeling tests

	Delamination strength (mN/mm)			
	Age ≤65 years Circ	Age ≤65 years Long	Age >65 years Circ	Age >65 years Long
Female	51 ± 7 (*n* = 9)	63 ± 9 (*n* = 9)	31 ± 6 (*n* = 10)	35 ± 7 (*n* = 10)
Male	55 ± 10 (*n* = 14)	60 ± 13 (*n* = 14)	42 ± 6 (*n* = 8)	49 ± 10 (*n* = 8)
*P*-Value	0.27	0.31	0.007	0.02

*P*-Values indicate statistical significance of the delamination strengths between Circ and Long directions.

Circ: circumferential; Long: longitudinal.

The computed dissection energy values in the circumferential and longitudinal peeling tests for males and females are summarized in Table 3. There were no statistically significant differences in the dissection energy between males and females for patients <65 years (circumferential: 10.6 ± 1.3 vs 11.5 ± 2.2 mJ/cm^2^, *P* = 0.31; longitudinal: 13.2 ± 2.0 vs 12.7 ± 2.6 mJ/cm^2^, *P* = 0.35). However, the dissection energy was significantly lower for females than that of males in the patient group larger than 65 years (circumferential: 5.8 ± 1.1 vs 7.8 ± 1.1 mJ/cm^2^, *P* = 0.02; longitudinal: 6.5 ± 1.3 vs 9.1 ± 2.2 mJ/cm^2^, *P* = 0.04).

**Table 3:** The computed dissection energy *W*^dissect^ (mJ/cm^2^) for the male and female ascending thoracic aortic aneurysm tissues in the circumferential and longitudinal peeling tests

	Dissection energy *W*^dissect^ (mJ/cm^2^)			
	Age ≤65 years Circ	Age ≤65 years Long	Age >65 years Circ	Age >65 years Long
Female	10.6 ± 1.3 (*n* = 9)	13.2 ± 2.0 (*n* = 9)	5.8 ± 1.1 (*n* = 7)	6.5 ± 1.3 (*n* = 7)
Male	11.5 ± 2.2 (*n* = 14)	12.7 ± 2.6 (*n* = 14)	7.8 ± 1.1 (*n* = 8)	9.1 ± 2.2 (*n* = 8)
*P*-Value	0.31	0.35	0.02	0.04

*P*-Values indicate statistical significance of the dissection energy values between Circ and Long directions.

Circ: circumferential; Long: longitudinal.

### Correlation between delamination strength and patient age

**Figure 4:**
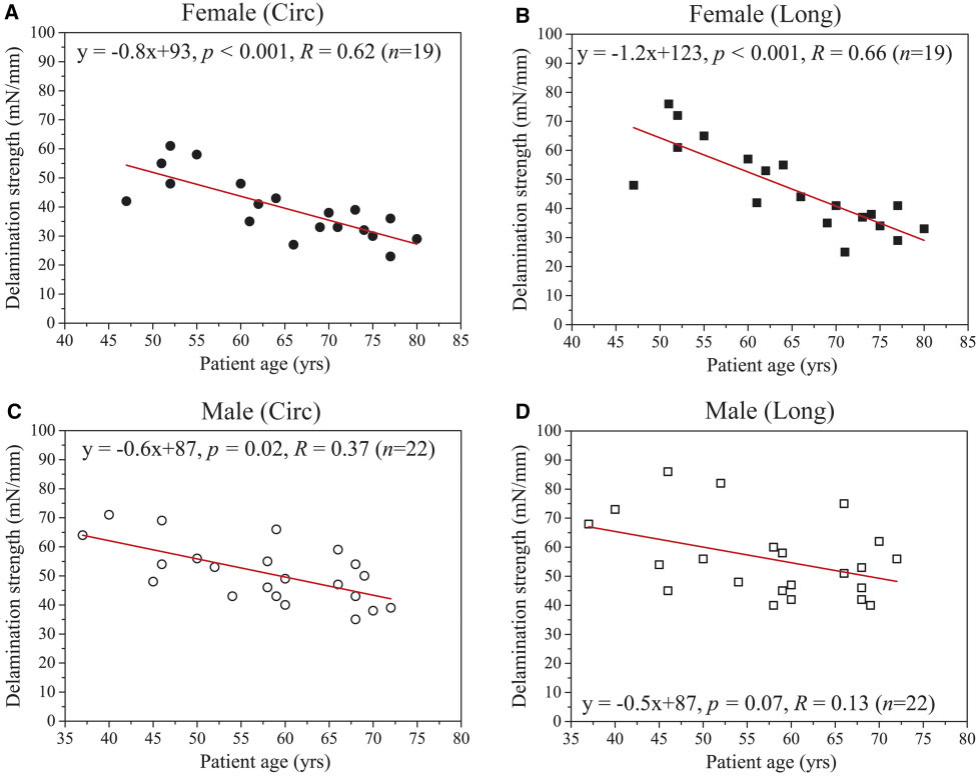
Scatter plots of the circumferential and longitudinal delamination strength of (**a** and **b**) female and (**c** and **d**) male ascending thoracic aortic aneurysms with respect to the related patient ages. Predictive equations obtained by linear regression model, associated with *p* and *R*, and number of specimens *n* are shown in each plot. The circumferential and longitudinal delamination strength values are significantly decreased and strongly correlated with the patient ages for females.

Scatter plots of the delamination strength with respect to the related patient ages are shown in Fig. 4. As can be seen from Fig. 4a and b, the delamination strength values in the circumferential and longitudinal directions were significantly decreased and strongly correlated with the patient ages for females (circumferential, *P* < 0.001, *R* = 0.62; longitudinal, *P* < 0.001, *R* = 0.66). However, the circumferential and longitudinal delamination strength values were not strongly correlated with the patient ages for males (circumferential, *P* = 0.02, *R* = 0.37; longitudinal, *P* = 0.07, *R* = 0.13; see Fig. 4c and d).

### Histology

**Figure 5:**
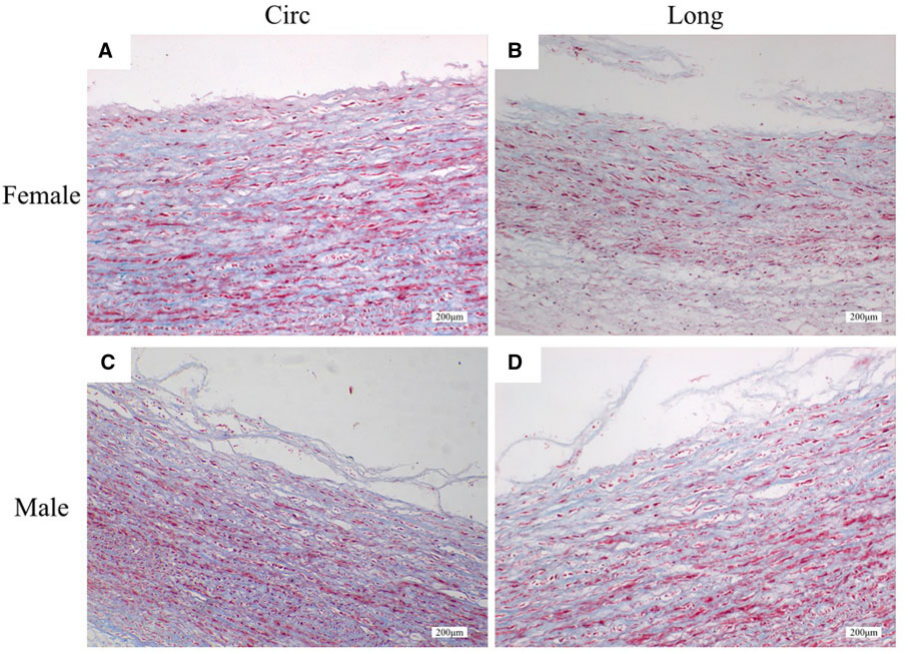
Representative histological images characterizing the morphology of the elderly (**a** and **b**) female and (**c** and **d**) male ascending thoracic aortic aneurysm tissues after the peeling tests. Dissection routes mainly propagate in the aortic media. The dissected surfaces in the circumferential peeling through the female ascending thoracic aortic aneurysms in (a) were much smoother than the longitudinal peeling in (b). (**c** and **d**) Both circumferential and longitudinal peeling tests of the male ascending thoracic aortic aneurysms generate rough dissected surfaces due to the rupture of collagen fibres in response to tearing. Circ and Long denote the circumferential and longitudinal directions, respectively. Original magnification 10×.

Representative histological images of the morphology of the dissected surfaces during the peeling tests are shown in Fig. 5. Dissection of the ATAAs primarily occurred in the aortic media for both males and females and the propagation routes were roughly parallel to elastic lamellae. The dissected surfaces in the circumferential peeling through the elderly females’ ATAAs were much smoother than the longitudinal peeling (Fig. 5a and b). For the elderly males’ ATAAs, however, both circumferential and longitudinal peeling tests may generate rough dissected surfaces due to the rupture of collagen fibres in response to tearing (Fig. 5c and d).

## DISCUSSION

It has been suggested that delamination strength is an important biomechanical indicator, which is helpful to assess the initiation and propagation of AD [6, 9, 10]. Thus, aortas that require lower forces to dissect may have a higher propensity of dissection. It is yet unclear whether gender differences exist in the delamination strength of ATAAs in the relatively young and elderly patients. In this study, we investigated gender-related dissection properties of the fresh ATAA tissue using peeling tests. More importantly, we established the correlations between delamination strength of the ATAAs and patient ages for both males and females.

Apparent oscillations in the peeling curves, as shown in Fig. 2, represent very unstable tearing of the tissue, even though a very low extension rate (i.e. 1 mm/min) is maintained. In particular, a higher mean force/width value is observed in the longitudinal peeling than in the circumferential peeling (Fig. 2c and d). It probably indicates that dissection properties of the ATAAs are anisotropic for some individual patients. A similar finding was noted in the dissection properties of human aortic media [18].

As can be seen from Fig. 3 and Tables 2 and 3, the circumferential and longitudinal delamination strength and dissection energy of the female ATAAs are statistically significantly lower than those of the males for the elderly patients. It suggests that there is a higher propensity of dissection occurrence for the elderly females when compared with the elderly males. This finding corroborates that the elderly females are more vulnerable to AD than the elderly males, which has been reported by Norton *et al.* [21]. An increased risk of AD for the elderly females could be due in part to the withdraw of protective effect of oestrogen on aorta [21]. When ATAAs have been diagnosed in the elderly females, physicians should be more aggressively to intervene the risk factors of AD, for instance, hypertension treatment and smoking cessation. The delamination strength values in the present study are much lower than a previous study, which investigated the effect of valve morphology on the dissection properties of ATAAs [9]. Our delamination strength values of the elderly female patients are similar to the dissection data of ATAAs measured by Chung *et al.* [10]. and Angouras *et al.* [22]. Note also that hypertension is a risk factor for AD. To confirm whether hypertension is a confounding factor that may affect the results, we have computed and compared the delamination strengths of hypertensive aortas (*n* = 20) and non-hypertensive aortas (*n* = 21). There are no statistically significant differences in the circumferential and longitudinal delamination strengths between hypertensive and non-hypertensive aortas (circumferential: 43 ± 8 vs 48 ± 9 mN/mm, *P* = 0.25; longitudinal: 50 ± 13 vs 57 ± 10 mN/mm, *P* = 0.29). Hence, hypertension is not a confounding factor in analysing the delamination strength of aneurysmal tissue in the present study.

As shown in Fig. 4a and b, the results suggest that there are strong correlations between the delamination strength and patient ages for females in both circumferential and longitudinal directions. However, this correlation is not present in the male patients, specifically for the longitudinal delamination strength (Fig. 4c and d). Hence, particular attention needs to be paid to elderly female patients in the clinical treatment of ATAAs as they may have a higher risk of AD initiation. Although a previous study indicated that delamination strength of the ATAAs was decreased as patient age increased [10], their dissection data were primarily derived from peeling through the longitudinal strips of aortic tissue. Note that some male patients with ages between 55 and 60 years have remarkably lower delamination strength for their ATAAs (Fig. 4c and d) and, thus, may also have a higher risk to initiate AD. To the authors’ knowledge, the present study is the first that shows the correlations between tissue delamination strength of the ATAAs and patient ages in different genders.

Dissection routes shown in histology (Fig. 5) can be attributed to the laminated structure of aortic media that is more prone to initiating a crack plane in parallel to elastic lamellae [23]. Similar locations of the dissected surfaces are also found for either healthy or aneurysmal aortas in the previous experimental studies [9, 18, 21, 24]. Notably, collagen fibre disruptions generated from the surfaces are observed in the circumferential and longitudinal peeling of the elderly male ATAAs (Fig. 5c and d). This feature may help explain why higher delamination strength is measured for the male ATAA tissues.

### Limitations

Some limitations of this study are summarized. First, all tissue samples were harvested from right lateral region of the aneurysms. In clinical observations, this region was associated with the high risk of dissection or rupture and was considered as the thinner region along the circumference of aorta due to the higher stresses generated by blood flow impingements [25]. However, regional distributions of delamination strength of the ATAAs remain unknown for male and female patients [22]. It may significantly affect the initiation and propagation of AD if the routes propagate across different regions. Second, peeling tests are performed under a prescribed extension rate of 1 mm/min. The protocol-controlled test is not representative of *in vivo* dissection propagation as spontaneous initiation of AD is more complex and multifactorial. However, biomechanical data in the present study are able to show the adhesive strength of aortic wall in resistance to propagating a dissection.

## CONCLUSION

For patients over 65 years, circumferential and longitudinal delamination strengths to peel apart the female ATAAs are statistically significantly lower than those of the males. Moreover, delamination strengths in the circumferential and longitudinal directions are significantly decreased and strongly correlated with the patient ages for females. These findings suggest that there is a higher propensity of AD occurrence for the elderly females when compared with the males. Therefore, surgeons should be cognizant of the risk of AD onset later in life, especially in females.

## ABBREVIATIONS

AD: Aortic dissection
ATAA: Ascending thoracic aortic aneurysm
PBS: Phosphate-buffered saline
